# Burnout in nursing during the COVID-19 pandemic: A bibliometric analysis of global research (2020–2023)

**DOI:** 10.1097/MD.0000000000046125

**Published:** 2026-01-02

**Authors:** Ya-Xi Lu, Da-long Wan

**Affiliations:** a Department of Geriatric Psychiatry, Huzhou Third Municipal Hospital, Huzhou, China; b Zhejiang University School of Medicine, Hangzhou, China; c Department of Hepatobiliary and Pancreatic Surgery, The First Affiliated Hospital, Zhejiang University School of Medicine, Hangzhou, Zhejiang, China.

**Keywords:** bibliometric, burnout, COVID-19, nurse

## Abstract

**Background:**

Burnout is an occupational phenomenon characterized by professionals experiencing a complete loss of concern and emotional connection towards the individuals they work with, resulting in their treatment in a detached or dehumanized manner. Extensive research has been conducted on burnout syndrome within the healthcare environment, however, prior to addressing this urgent public health issue, it is crucial to examine the existing literature on burnout among nurses during the COVID-19 pandemic and identify relevant variables explored in recent articles.

**Methods:**

An extensive literature search was conducted in the Web of Science Core Collection database to identify all relevant studies on nursing burnout during the COVID-19 pandemic.

**Results:**

According to the search strategy, a total of 1051 eligible publications were collected from the time of January 01, 2020 to December 31, 2023 in Web of Science Core Collection database. Finally, 946 eligible publications, including 865 articles and 81 reviews, were included in the subsequent analysis. In the inaugural year of the COVID-19 pandemic in 2020, a mere 48 articles were dedicated to examining the correlation between the pandemic and nursing staff burnout. However, this figure surged to 215 publications in 2021 and further escalated to an impressive count of 380 articles in 2022.

**Conclusion:**

In summary, this is the first comprehensive analysis of publications related to nursing burnout in the context of COVID-19 from 2020 to 2023 through bibliometrics. Our results show the COVID-19 pandemic has certainly had a significant impact on the occupational burnout of nurses.

## 1. Introduction

Burnout is an occupational phenomenon characterized by professionals experiencing a complete loss of concern and emotional connection towards the individuals they work with, leading to their treatment in a detached or dehumanized manner.^[1]^ The World Health Organization’s (WHO) International Classification of Diseases, 11th Revision, has recently recognized burnout as an “occupational phenomenon.” According to WHO, burnout is defined as a syndrome that results from poorly managed workplace stressors leading to physical symptoms such as sleep disorders, anxiety, and a decreased ability to complete work-related tasks.^[2]^ Healthcare workers (HCWs) consistently face emotionally draining stressors while providing complex care and treatment to patients, thereby increasing the risk of occupational burnout. The nursing workforce constitutes over 60% of the healthcare industry and is widely recognized as its cornerstone, with nurses exhibiting a higher prevalence of burnout.^[3]^ The susceptibility of nurses to contracting communicable diseases is heightened due to their exposure to blood, body fluids, and airborne microbes. Moreover, their direct patient care activities result in a higher frequency of encounters with communicable diseases.^[4]^ Burnout significantly impacts nurses’ physical and mental health, as well as the quality of care, and patients’ recovery. It manifests in physical symptoms like fatigue, headaches, and insomnia, along with reduced concentration and memory capacity. Consequently, it leads to various forms of mental illness including anxiety, distress and even suicidal ideation.^[5]^ Alarmingly, the WHO estimated a global deficit of approximately 7.6 million nurses by 2030.^[6]^ Nurses exhibited a significantly elevated suicide rate in comparison to the general population with a history of mental health issues.^[7]^ Undoubtedly, the outbreak of the COVID-19 pandemic has exacerbated the situation.

The coronavirus is characterized, in part, by respiratory illness and can be transmitted through various means, including direct contact, indirect contact, as well as airborne transmission. After 4 years since its outbreak began, COVID-19 continues to undergo evolutionary changes. Consequently, it has infected numerous healthcare professionals and exerted a substantial impact on global healthcare systems that are already overwhelmed.^[8]^ During the peak of the COVID-19 pandemic, nurses across all sectors of the profession assumed diverse roles that have contributed to the achievements witnessed in combating the pandemic. Consequently, nurses have reported an increase in rates of mental and emotional distress during pandemics, thereby impacting their capacity to uphold well-being amidst the COVID-19 crisis.^[9,10]^ Despite the extensive research highlighting burnout as a prevalent concern among nurses, exacerbated by the COVID-19 outbreaks, there remains an incomplete understanding of both the risk factors and protective measures associated with burnout.^[11]^ Van Mol et al highlighted that working in a very stressful environment can have a detrimental effect on a medical professional’s overall health and wellbeing. Nevertheless, how burnout is associated with anxiety and depression remains unclear.^[12]^ In fact, adequate support nurses’ mental health being with respect to the local context has been recommended as a key to maintaining and strengthening of public health capacities.^[13]^

Currently, a variety of scientific methodologies are employed to comprehensively comprehend the present state of an academic discipline, with bibliometric analysis utilizing mathematical and statistical techniques emerging as the predominant approach for obtaining a holistic understanding of knowledge structure and research priorities within an academic domain.^[14,15]^ Therefore, it is imperative to conduct studies that facilitate mapping and analysis of scientific output pertaining to burnout among nurses, particularly in emergency situations or outbreaks. The analysis of scientific literature production can provide valuable data and information on a specific topic. Bibliometrics serves as an essential tool for evaluating the development of a particular subject, based on its intellectual and societal contributions. In addition to describing the current research landscape, bibliometrics enables the assessment of research prospects and prediction of emerging trends in academic inquiry, surpassing other methods such as meta-analyses, conventional reviews, or experimental studies.^[16]^ Unlike previous studies that either conducted direct surveys of nurses’ burnout experiences during the COVID-19 pandemic or performed bibliometric analyses of nursing burnout in general, our study is the first to conduct a global bibliometric analysis of published literature specifically examining nursing burnout during the COVID-19 pandemic (2020–2023). This pandemic-centered, global approach provides a broader and more integrated perspective on the scientific output and emerging research trends. Therefore, our research aims to identify scientific contributions concerning burnout among nursing professionals amidst the COVID-19 pandemic, while analyzing their origins in terms of countries/institutions, scientific journals, authors, keywords, citations, and emerging trends.

## 2. Methods

### 2.1. Database and searching strategy

**Figure 1.**
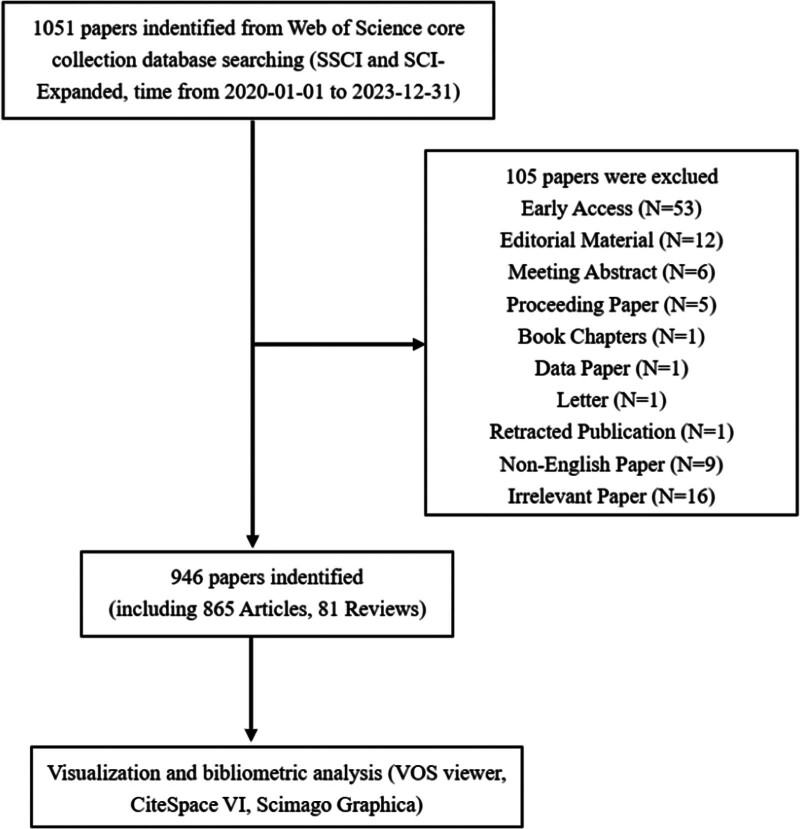
Detailed flowchart of search.

We conducted an extensive literature search in the Web of Science Core Collection (WoSCC) database to identify all relevant studies on nursing burnout during the COVID-19 pandemic. The WoSCC by Clarivate Analytics is a highly regarded and comprehensive interdisciplinary database, widely recognized as a prominent data source for bibliometric studies. It encompasses an extensive collection of academic journals and literature. The search process was conducted independently by 2 reviewers according to the following inclusion and exclusion criteria: publications were sourced from the WoSCC Science Citation Index Expanded or Social Sciences Citation Index databases. Only publications in English were considered. The publication types were limited to original articles and reviews. Various other publication types such as meeting abstracts, editorial materials, early access papers, proceeding papers, book chapters, letters, corrections, and news items were excluded. The retrieval time spanned from January 01, 2020 to December 31, 2023. The subjects of the publications focused on nurses. Literature exclusively focusing on other healthcare professionals, such as physicians or medical volunteers, was excluded. The databases were used to retrieve the relevant publications using the following search string: TS = (burnout) AND TS = (nurse or nursing) AND TS = (covid-19). Finally, 946 eligible publications, including 865 articles and 81 reviews, were included in the subsequent analysis (Fig. 1).

### 2.2. Data analyses and visualization

In this study, The WoSCC intrinsic toolkits were used to analyze general characteristics of eligible literatures, including the list of Web of Science subject categories, the number of annual publications, citations and the h-index. The bibliometric analysis and visualization were performed by CiteSpace (Version 6.2.6; Drexel University, Philadelphia), VOSviewer (Version 1.6.20; Centre for Science and Technology Studies, Leiden University, Leiden, The Netherlands) and a free desktop application (Scimago Graphica, SCImago Lab, Albolote, Spain). VOSviewer is a wildly applied bibliometric analysis tool, which provided 3 kinds of visualization maps including the network visualization, the overlay visualization and the density visualization. Scimago Graphica is a desktop application designed to analyze and visualize data. CiteSpace is another citation visualization analysis software developed by Chen et al. There is currently no consensus regarding the optimal approach for bibliometric analysis. Therefore, we employed a combination of VOSviewer, Scimago Graphica, and CiteSpace to leverage their respective properties and advantages in our subsequent analysis.

The H-index was used to assess both productivity and citation impact. Total link strength, as calculated by VOSviewer, quantified the overall strength of an entity’s collaborative links. Burst detection, as implemented in CiteSpace, identified keywords and references that exhibited sudden increases in frequency, reflecting emerging trends and research frontiers.

For the purpose of network visualization, specific thresholds were applied. For co-citation analysis, a minimum threshold of 100 was adopted. For author co-authorship analysis, the inclusion criterion was ≥3 publications. For institutional analysis, institutions with ≥6 publications were included, while for keyword co-occurrence, only keywords appearing at least 40 times were analyzed. These thresholds were chosen in line with commonly adopted practices in bibliometric studies. Setting lower thresholds resulted in excessively dense and less interpretable networks, whereas higher thresholds risked excluding relevant nodes. The selected values ensured a balance between inclusiveness and interpretability.

In this research, when calculating the countries and districts of origin of the indexed documents, documents from China included those from Taiwan, Hong Kong, and Macao. We mainly adopted VOSviewer and Scimago Graphica to conduct author-keywords co-occurrence analysis, co-authorship analysis of countries/regions, authors, institutions, and co-citation analysis of journals or references. CiteSpace was utilized to accomplish co-citation analysis of authors and references, dual-map overlay of journals and citation burst of keywords or references.

### 2.3. Ethical review

As this study analyzed secondary data from published articles indexed in the Web of Science Core Collection, ethical approval and informed consent were not required.

## 3. Results

### 3.1. Document type and quantity

According to the search strategy, a total of 1051 eligible publications were collected from the time of January 01, 2020 to December 31, 2023 in WoSCC database. Subsequently, 105 literatures were extracted, including Early Access (N = 53), Editorial Material (N = 12), Meeting Abstract (N = 6), Proceeding Paper (N = 5), Book Chapters (N = 1), Data Paper (N = 1), Letter (N = 1), Retracted Publication (N = 1), Non-English Paper (N = 9), Irrelevant Papers were removed manually (N = 16). Finally, 946 eligible publications, including 865 articles and 81 reviews, were included in the subsequent analysis. In the inaugural year of the COVID-19 pandemic in 2020, a mere 48 articles were dedicated to examining the correlation between the pandemic and nursing staff burnout. However, this figure surged to 215 publications in 2021 and further escalated to an impressive count of 380 articles in 2022. Nevertheless, due to the relaxation of epidemic prevention and control measures across diverse nations, there was a gradual decline observed in the number of articles on this subject matter in 2023, with a total tally of 303 publications. It is worth noting that this decrease partly stems from certain journal articles published in December not being promptly incorporated into the WoSCC database.

### 3.2. Contributions of countries/regions

**Figure 2.**
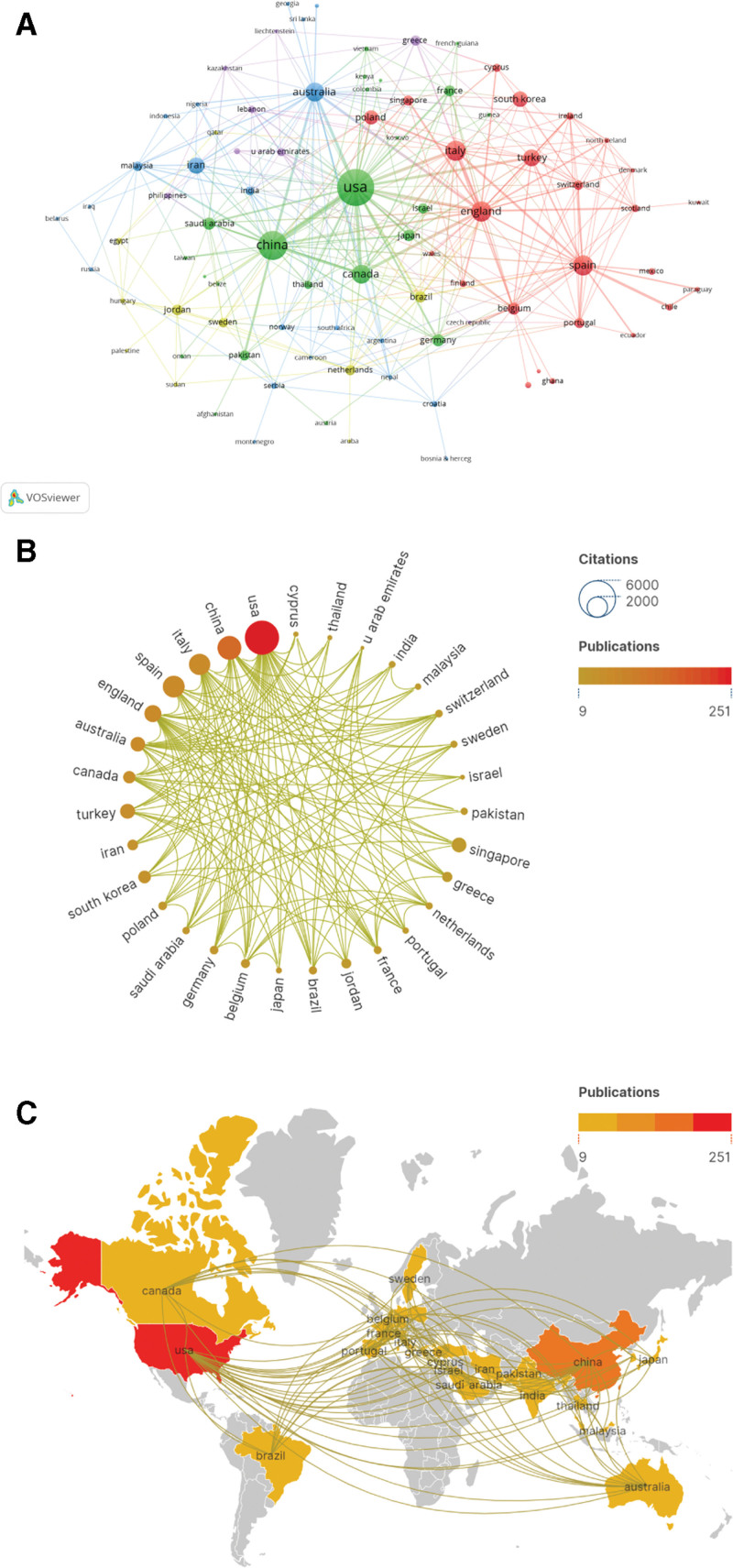
(A) The countries/regions citation overlay visualization map generated by using VOS viewer. (B) The cross-country/region collaborations visualization map. (C) Geographic distribution map based on the total publications of different countries/regions.

In the past 4 years, a total of 2094 research institutions from 91 countries had contributed in the field of nursing burnout. Figure 2A showed the collaboration map of all of 91 countries. The top 10 productive countries in this field were listed in Table 1, revealing the United States as the leading country with 251 publications (26.52%), surpassing China (143, 15.12%) by 88 articles. Furthermore, the total number of citations for the United States far exceeded that of any other country and was more than twice that of China, indicating its position as a cutting-edge leader in this field worldwide. However, when considering average citations, Spain, Italy, and Turkey emerged as the top 3 countries whose articles are likely to be widely recognized. Information from the published articles was used to create a world map illustrating the cooperative relationship of the research topic in top 30 different countries. The international cooperation analysis is presented in Figure 2B. The size of the circle corresponds to the number of citations, while the color intensity indicates the number of publications. The thickness of the lines connecting countries reflects the frequency of collaboration. The figure reveals a limited level of collaboration between the United States, which holds the highest position in terms of article publications, and China, ranking second. Furthermore, it highlights that the United States demonstrates a stronger inclination towards partnering with European countries, Australia, Japan, and South Korea. The world map in Figure 2C showed that publications in this field were mainly published by countries from America, Asia, Europe. Meanwhile, the close alignment of cooperation among productive countries/regions was evident.

**Table 1:** Top10 productive countries related to nursing burnout during the COVID-19 research.

Rank	Country	Documents	Citations	Average citation	Total link strength
1	USA	251	4701	18.73	54
2	China	143	2284	15.97	38
3	Spain	65	1833	28.20	24
4	Italy	63	1930	30.63	16
5	England	61	1168	19.15	35
6	Turkey	55	861	15.65	26
7	Australia	53	614	11.58	27
8	South Korea	43	899	20.91	13
9	Canada	40	463	11.58	7
10	Iran	34	656	19.29	4

### 3.3. Distribution of institutions

**Figure 3.**
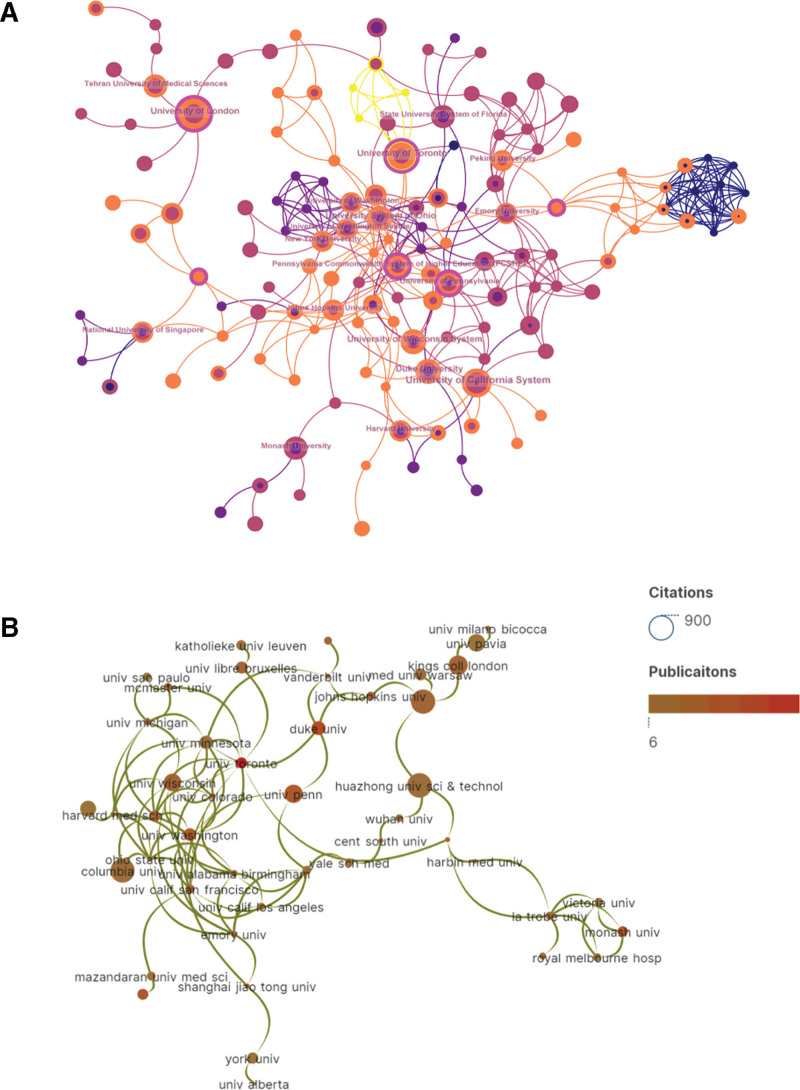
(A) The visualization map of institutions analysis generated by CiteSpace. (B) The linkage of institutions viewed by Scimago Graphica.

The field was significantly enriched by the contributions of 2094 research institutions. Table 2 presents a comprehensive overview of the top 10 research institutions that made notable contributions. Notably, 4 out of these leading organizations with the highest publication count are affiliated with prestigious institutions in the United States. This observation implies that American agencies prioritize addressing nurses’ burnout amidst the COVID-19 pandemic to a greater extent. The University of Toronto stands out as the largest institution in terms of publication count. However, it is worth noting that these 16 articles have an average citation rate that does not rank significantly high (value = 9.63). On the other hand, Singapore’s National University distinguishes itself by achieving remarkably higher average citations (value = 85.22) compared to those of other organizations. The map of institutions analysis generated by CiteSpace was depicted in Figure 3A. Furthermore, the findings revealed that each country exhibited stronger affiliations with its own institutions and weaker connections with foreign institutions, resulting in a more pronounced presence of forks in the overall chart. In order to better observe the relationship between institutions, we selected 46 institutions with a minimum of 6 publications and contacts for visualization processing. Figure 3B the linkage of these 46 institutions using Scimago Graphica. The low junction of institutions network indicates the lack of interagency collaboration and further emphasizes the importance of collaboration.

**Table 2:** Top 10 institutions ranked by the numbers of publications.

Rank	Organization	Countries	Documents	Citations	Average citation
1	University of Toronto	Canada	16	154	9.63
2	Duke University	USA	13	254	19.54
3	University of Washington	USA	11	203	18.45
4	Tehran University of Medical Sciences	Iran	11	155	14.09
5	University of Pennsylvania	USA	10	434	43.40
6	Monash University	Australia	10	141	14.10
7	National University of Singapore	Singapore	9	767	85.22
8	King’s College London	England	9	478	53.11
9	University of Wisconsin System	USA	9	403	44.78
10	University of Huelva	Spain	9	360	40.00

### 3.4. Analysis of top journals and co-cited journals

**Figure 4.**
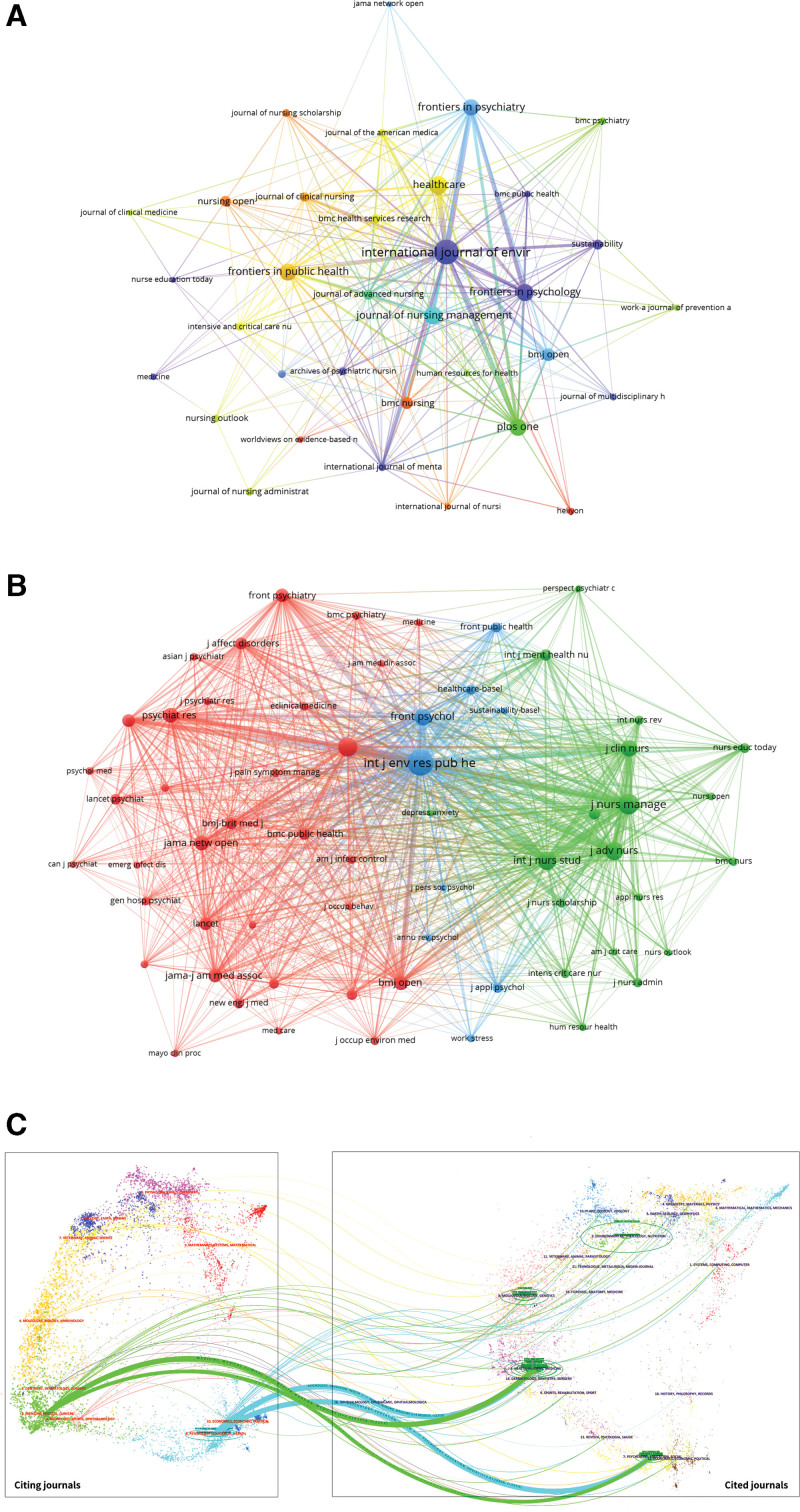
(A) Network visualization map of Journal analysis generated by VOSviewer. (B) Network visualization map of Journal co-cited analysis generated by VOSviewer. (Connecting lines represent collaboration between Journals, and the same color of node represent the same cluster.) (C) The dual-mapping overlap of journals on research related to nursing burnout in the context of COVID-19 was conducted using CiteSpace.

At present, the research papers related to nursing burnout during the COVID-19 had been published in 283 scholarly journals. From the results of Table 3, the journal International Journal of Environmental Research and Public Health had the highest number of publications, with a total citations of 1662 times, followed by Healthcare and Journal of Nursing Management. It is evident that the top 10 journals in terms of publication volume are exclusively open access journals, with some being classified as predatory. For instance, the International Journal of Environmental Research and Public Health, which holds the highest rank in terms of publication volume, is not indexed by SCI database. In general, nursing journals exhibit a relatively low impact factor (IF), with only 2 out of the top 10 journals surpassing an IF of 5. The remaining 7 journals all have scores below 5, indirectly suggesting a comparatively limited level of attention given to articles within the field of nursing. According to the Journal Citation Report for 2020, 80% of these journals were classified in Q1/Q2. However, this does not diminish the substandard quality of the top 10 magazines, as Frontiers in Psychology had the highest average citation count of only 22.67. In this study, a total of 34 journals were identified that published >5 papers. To visualize the interconnections among these journals, we employed VOSviewer software to generate a journal network map (Fig. 4A).

**Table 3:** Top 10 journals with the highest number of articles.

Rank	Journal title	Publications	Citations	Average citation	IF (2022)	JCR (2022)
1	International Journal of Environmental Research and Public Health	81	1662	20.52	/	Q2
2	Healthcare	44	337	7.66	2.8	Q3
3	Journal of Nursing Management	41	494	12.05	5.5	Q1
4	Frontiers in Psychology	39	884	22.67	3.8	Q2
5	Frontiers in Public Health	37	140	3.78	5.2	Q2
6	Plos One	37	677	18.30	3.7	Q1
7	Frontiers in Psychiatry	36	584	16.22	4.7	Q2
8	BMJ Open	21	446	21.24	2.9	Q1
9	BMC Nursing	19	55	2.89	3.2	Q2
10	Nursing Open	16	106	6.63	2.3	Q1

IF = Impact Factor, JCR = Journal Citation Reports.

Journal co-citation is a significant index to reflect the influence of a journal. In this study, 62 journals had been co-cited at least 100 times and we used VOSviewer to generate a journal co-cited network map (Fig. 4B). As shown in Figure 4B, the top 3 journals with the highest total link strength were International Journal of Environmental Research and Public Health, Journal of Nursing Management, and Plos One. Figure 4C was a dual-map, which was used to represent the discipline distribution of journals involved in research, and through this method, we could clearly understand the knowledge flows among different disciplines and the frontier or hotspot of each discipline. It could be found that the literature published in Psychology/Education/Health or Medicine/Medical/Clinical journals often cited the literature from Psychology/Education/Social or Health/Nursing/Medicine journals.

### 3.5. Analysis of the active authors and co-cited authors

**Figure 5.**
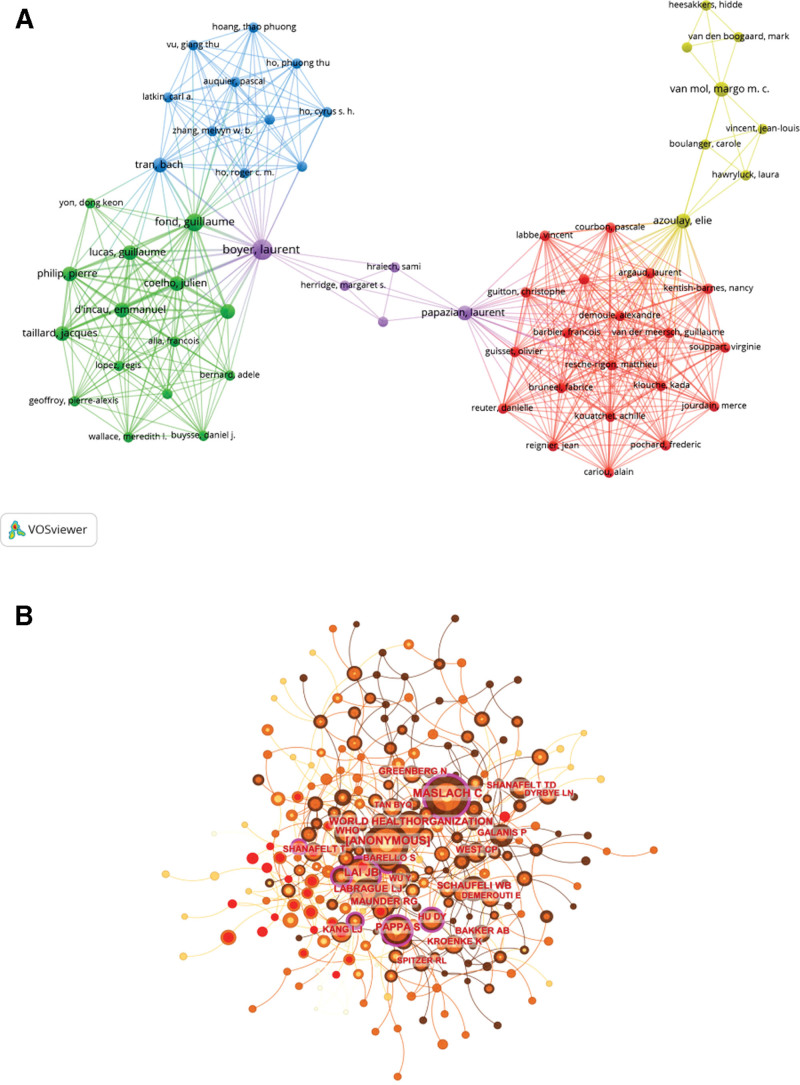
(A) Co-authorship network visualization map of authors. Circles represent the number of articles published. Connecting lines represent collaboration between authors, and the same color of node represent the same cluster. (B) Co-citation network visualization map of authors produced by CiteSpace.

A total of 5222 authors participated in the publication of papers addressing nursing burnout in the context of COVID-19. Table 4 summarized the top 10 most productive authors and the top 10 co-cited authors. The top 10 most productive authors were from the United States, Canada, Australia, and European countries. Rounding out the top 3 were Bruyneel A (5 articles, 110 citations) from Belgium, Pascoe A (5 articles, 83 citations) from Australia, and Linzer M (4 articles, 231 citations) from the United States. In terms of average citations, the top 3 are Linzer M (USA), Giostra V (Italy), and Wahlste S (USA). Authors were visualized using VOSviewer software and showed in Figure 5A. Meanwhile, the collaboration relationship among these authors were displayed. As shown in Figure 5A, Papazian L, Laurent B, Azoulay E, Boyer L, Fond G, and Tran B were key authors connecting multiple research clusters. However, overall, there was little collaboration and communication among various research clusters. Through co-citation analysis of authors, we found that the total citations of Maslach C, WHO and Lai JB ranked in the top 3. Maslach C is the creator of the Maslach Burnout Inventory (MBI) and the WHO is the world’s official institution, with Lai JB from China being the first to report on the impact of COVID-19 on the mental health of HCWs. The co-citation analysis of authors was performed by CiteSpace software (Fig. 5B). The results demonstrated that Maslach C was at the center of the research in this field.

**Table 4:** The 10 most productive authors and top 10 co-cited authors.

Rank	Author	Country	Documents	Citations	Co‑cited author	Country	Citations
1	Bruyneel A	Belgium	5	110	Maslach C	USA	452
2	Pascoe A	Australia	5	83	WHO	/	360
3	Linzer M	USA	4	231	Lai JB	China	225
4	Giostra V	Italy	4	225	Labrague L	Oman	152
5	Wahlste S	USA	4	163	Pappa S	England	149
6	Smith P	Belgium	4	102	Shanafelt T	USA	145
7	Galiana L	Spain	4	88	Schaufeli W	Belgium	142
8	O’Brien EC	USA	4	53	Maunder R	Canada	127
9	Heeney ND	Canada	4	52	West C	USA	110
10	Boamah SA	Canada	4	38	Barello S	Italy	99

WHO = World Health Organization.

### 3.6. Analysis of co-cited references

**Figure 6.**
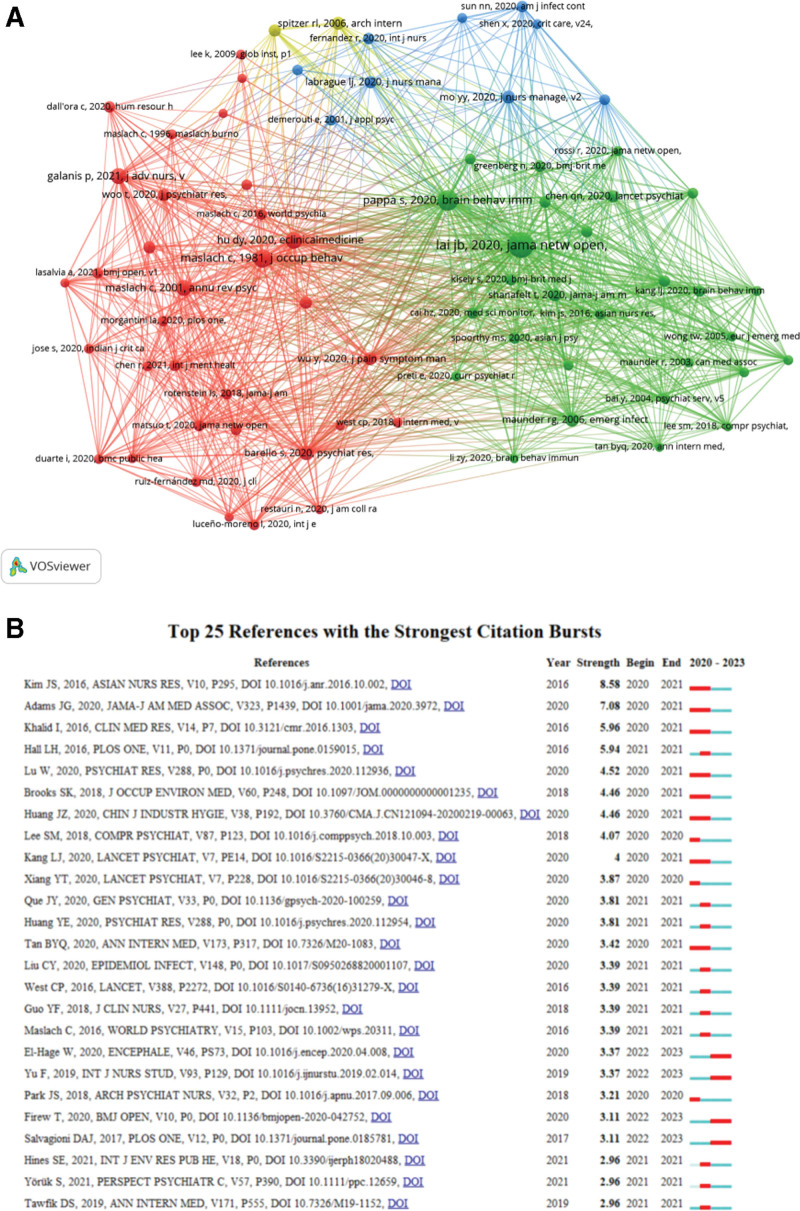
(A) Network visualization map of Cluster view. (B) Visualization map of top 25 references with the strongest citation bursts.

The number of citations is a measure of impact of a publication in a scientific field. A total of 27,711 papers were included in this study, of which 152 publications were cited >20 times. Table 5 listed the top 10 cited paper.^[1,17–25]^ The most cited article was the article published by Lai JB in 2020, with a total of 225 times, followed by Pappa S (2020) and Maslach C (1981), with 136 and 91 times, respectively. It is noteworthy that 2 of the top 10 highly cited articles were authored by Maslach C, aligning with the previously most cited authors in this field. One article provides a comprehensive definition of burnout, while the other presents a standardized measurement scale for assessing burnout levels. These articles are considered essential classics for conducting research on burnout. Figure 6A illustrates the interrelationships among the subset of 152 articles that have been cited >20 times. The top 25 references exhibiting the strongest citation bursts were summarized in Figure 6B. Among them, the paper on new deep residual nets published by Kim JS et al in 2016 demonstrated the highest strength value.^[26]^ Subsequently, an article authored by Adams JG et al published in the JAMA-Journal of the American Medical Association in 2020, exhibited a burst intensity value of 7.08.^[27]^ In third place was Khalid I’s study titled “Healthcare Workers Emotions, Perceived Stressors and Coping Strategies During a MERS-CoV Outbreak” from 2016.^[28]^ The reference citation burst for this investigation commenced in 2020 and has persisted until present with its latest detection recorded in 2023. Currently, it is observed that most of the top 10 literature regarding outbreak intensity originated between 2020 and concluded by 2021, indicating heightened interest during COVID-19’s early stages. However, as our understanding of this novel coronavirus epidemic gradually improved over time, research on nursing burnout subsequently declined along with diminishing hotspots.

**Table 5:** The top 10 articles most cited in research on nursing burnout during the COVID-19 pandemic.

Title	Journal	First author	Year	IF (2022)	Citations
Factors associated with mental health outcomes among health care workers exposed to coronavirus disease 2019	JAMA Network Open	Lai JB	2020	13.8	225
Prevalence of depression, anxiety, and insomnia among healthcare workers during the COVID-19 pandemic: A systematic review and meta-analysis	Brain Behavior and Immunity	Pappa S	2020	15.1	136
The measurement of experienced burnout	Journal of Organizational Behavior	Maslach C	1981	6.8	116
Psychiatric disorders among hospitalized patients deceased with COVID-19 in Italy	EClinicalMedicine	Hu DY	2020	15.1	91
Job burnout	Annual Review of Psychology	Maslach C	2001	24.8	88
Nurses’ burnout and associated risk factors during the COVID-19 pandemic: A systematic review and meta-analysis	Journal of Advanced Nursing	Galanis p	2021	3.8	86
Burnout and somatic symptoms among frontline healthcare professionals at the peak of the Italian COVID-19 pandemic	Psychiatry Research	Barello s	2020	11.3	74
Long-term psychological and occupational effects of providing hospital healthcare during SARS outbreak	Emerging Infection Diseases	Maunder RG	2006	11.8	64
A comparison of burnout frequency among oncology physicians and nurses working on the frontline and usual wards during the COVID-19 epidemic in Wuhan, China	Journal of Pain and Symptom Management	Wu Y	2020	4.7	60
A brief measure for assessing generalized anxiety disorder: the GAD-7	Archives of Internal Medicine	Spitzer RL	2006	/	58

SARS = severe acute respiratory syndrome.

### 3.7. Keywords co-occurrence analysis

**Figure 7.**
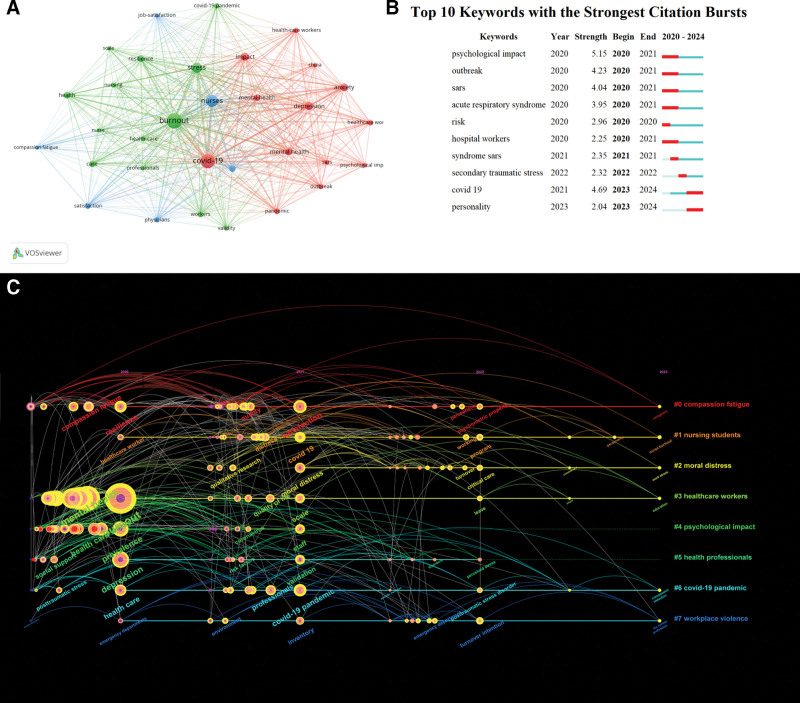
(A) The overlay visualization map of author keywords co-occurrence analysis. (B) Visualization map of top 10 keywords with the strongest citation burst. (C) The timeline view of keywords.

A total of 2235 author keywords were included in this study. The correlation between the top 32 keywords, appearing at least 40 times, is depicted in Figure 7A. The frequency of the leading 10 keywords includes burnout, COVID-19, nurses, stress, impact, depression, anxiety, mental health, prevalence, and resilience (all of which align with our study focus). Notably significant citation bursts were observed for the top 10 keywords as summarized in Figure 7B. Based on the keywords depicted in the figure, it is evident that during the period spanning 2020 to 2021, due to the nascent stage of awareness regarding the novel coronavirus epidemic, frequent comparisons were made with the severe acute respiratory syndrome epidemic, also known as severe acute respiratory syndrome. The top 5 emerging terms exhibit a similar temporal pattern as observed in references bursts, commencing in 2020 and concluding in 2021. Regarding the inclusion of 2024 among burst words, this can be attributed to our search deadline being December 31st, 2023. Consequently, certain articles from December 2023 are recognized by CiteSpace as pertaining to 2024. The timeline view of keywords is a visual diagram that can reflect the temporal characteristics of the research hotspots in this field (Fig. 7C). Among the 7 clusters, #0 compassion fatigue was the earliest research hotspot in this field, and had been studied until recent years. The majority of the keywords emerged in 2020. Notably, the most prominent keywords observed in 2020 are #4 psychological impact and #3 HCWs. However, the frequency of occurrence for the keyword cluster associated with #4 has exhibited a decline since 2022, whereas the keyword pertaining to #3 HCWs continues to persist until present.

## 4. Discussion

Since the identification of the COVID-19 outbreak in Wuhan, China, in December 2019, a surge of articles on this topic emerged in January 2020.^[29–32]^ It has been over 4 years since the detection of the coronavirus outbreak. The first article discussing the mental health of medical staff during the COVID-19 pandemic was published by Lai JB.^[33]^ Our search strategy yielded a total of 946 articles on occupational fatigue among nursing staff during this pandemic, comprising 865 research papers and 81 literature reviews. Furthermore, we employed bibliometric analysis to investigate job burnout among nursing staff using a similar methodology as de Oliveira et al.^[34]^ Additionally, various authors conducted country-specific analyses on job burnout among nursing staff.^[35]^ However, our study is pioneering in conducting a bibliometric analysis of literature on burnout among nursing staff during the novel coronavirus epidemic with an aim to identify scientific contributions in this area amidst the ongoing COVID-19 pandemic. This analysis helps researchers stay informed about current hotspots and emerging trends in this research field by summarizing its development and analyzing emerging trends.

According to the results of countries/regions distribution, among the 91 countries/regions involved in this study, the United States (251, 26.52%) was the country with the largest number of published articles, followed by China (143, 15.12%), which together accounted for 41.62% of all papers, demonstrating their leadership in this field. The aforementioned factors are closely associated with the United States’ possession of cutting-edge scientific research equipment and the largest pool of scientific researchers globally, meanwhile, China emerged as the first nation to officially document the outbreak. However, in terms of average citations, Spain and Italy emerged as the top 2 countries whose articles are likely to be widely recognized. Figure 2B reveals a limited level of collaboration between the United States, which holds the highest position in terms of article publications, and China, ranking second. Furthermore, it highlights that the United States demonstrates a stronger inclination towards partnering with European countries, Australia, Japan, and South Korea. This collaboration can be attributed, in part, to disparities in COVID-19 prevention policies between the United States and China.

Figure 3A reveals a tendency for close cooperation among several institutions in specific areas relatively speaking, whereas Figure 3B provides a more intuitive representation with relatively fewer connecting lines indicating that most research collaborations and communication were limited to certain institutions in North America, Europe, and select Asian countries. Therefore, enhancing international collaboration, particularly with institutions in developing countries, would be beneficial. Identification of prominent journals and conducting journal co-citation analysis can furnish researchers with a plethora of dependable reference information, aiding them in determining the most appropriate target journals during literature search or research submission.^[36]^ Besides total citations, IF and Journal Citation category serve as 2 pivotal indicators for assessing the scholarly standing of journals.^[37,38]^ The top 10 journals in terms of publication volume, as depicted in Table 3, predominantly encompass 3 categories: public health journals, mental health journals, and nursing journals. This alignment is consistent with the subject matter of our study. COVID-19 falls within the realm of public health, while our research focuses on mental health and specifically targets nursing staff. Consequently, articles pertaining to our topic are primarily disseminated through these 3 prominent journals. Certainly, certain journals listed in the table may be classified by scholars as predatory journals due to their extensive publication output. Journals that are often classified as predatory typically exhibit several problematic features, such as inadequate reporting of research methods, lack of ethical approval for studies, evidence of plagiarism, and the absence of rigorous peer-review processes. These practices collectively undermine the credibility and reliability of the scholarly literature.^[39]^ For example, the *International Journal of Environmental Research and Public Health* was delisted from the Science Citation Index database in 2023 because of its excessively high publication volume and elevated self-citation rates, both of which are commonly cited indicators of questionable editorial practices. Such patterns suggest that the journals in question may prioritize financial or editorial gain over scientific integrity. As a result, their publications risk compromising the credibility of nursing research and may have adverse implications for clinical practice.^[40]^ In fact, it is worth discussing the relatively low overall IF of nursing journals. The highest IF among all nursing journals in 2022, based on Web of Knowledge data, was 11.20 (International Journal of Nursing Studies). Out of a total of 130 nursing journals, only 11 had an IF exceeding 5. Researchers bear responsibility for ensuring the quality and credibility of papers published in these self-identified “nursing” journals, both for the benefit of the nursing field and the general public. Given the criticism surrounding IFs and the absence of effective alternative standards to assess quality, it may be timely for nursing to devise strategies that demonstrate how published research in this domain enhances clinical outcomes and consumer care.^[41]^

In the analysis of author co-authorship, it is noteworthy that 5 out of the top 10 authors hailed from North America, with 3 being from the United States and 2 from Canada. Additionally, 4 authors were affiliated with institutions in Europe, while one author represented Oceania. This observation suggests that caregivers in economically developed countries possess a relatively advantageous position for conducting burnout-related research. Notably, Bruyneel A emerged as a prominent contributor who extensively investigated paramedic burnout during the COVID-19 pandemic in Belgium.^[42,43]^ Figure 5A reveals key authors such as Papazian L, Laurent B, Azoulay E, Boyer L, Fond G, and Tran B acting as crucial connectors between multiple research clusters (a plausible explanation for their high citation rates). However, it is worth noting that there appears to be limited collaboration among different research teams based on the scarcity of connections between distinct colors or clusters. In terms of author co-citation analysis, Maslach C, WHO, and Lai JB emerged as the top 3 influential figures. Maslach C is a renowned authority in the field of burnout research, and their comprehensive approach to assessing burnout involves utilizing psychometric variables from a scale developed by Maslach and their colleague Jackson, known as the Maslach Burnout Inventory (MBI).^[21]^ The MBI has gained widespread recognition in the literature as the gold standard for assessing burnout levels. It encompasses a self-administered scale comprising affirmative statements that capture individuals’ work-related feelings and attitudes. Since its initial publication in 1981, the MBI has undergone progressive updates, translations, and adaptations to cater to the diverse needs of professional groups worldwide.^[44]^

Citation analysis and co-citation analysis of references are crucial methodologies in bibliometric studies, employed to identify seminal literature, assess the progression of research, and forecast emerging frontiers. Highly cited articles typically represent high-quality research characterized by strong innovation and significant impact within a specific field. Table 4 presents the top ten most frequently cited studies. Lai JB, previously the third most cited author, his article published in JAMA Network is the most cited article. This study revealed that HCWs responding to the COVID-19 outbreak reported significantly elevated rates of depressive symptoms, anxiety, insomnia, and distress. Urgent implementation of targeted interventions aimed at promoting mental wellness among HCWs exposed to COVID-19 is warranted, with particular attention given to women, nurses, and frontline personnel.^[23]^

Burst detection is an algorithm developed to capture sharp increases in the popularity of references or keywords within a specific timeframe, serving as an efficient method for identifying hotspots or topics. Our findings suggest that the initial burst of reference citations in this field revolved around factors influencing burnout among emergency nurses during the outbreak of Middle East Respiratory Syndrome Coronavirus (MERS) in Korea, as published by Kim JS et al Their study concluded that emergency nurses caring for MERS-CoV-infected patients should be aware of higher burnout levels compared to nurses in other hospital departments, with job stress being the primary influential factor. This implies that regardless of whether it is MERS or COVID-19, caregiver burnout remains a challenging issue worthy of contemplation.

Co-occurrence analysis of keywords is a common method used in bibliometrics to identify popular research topics, which can reflect the changing process of research topics in the whole field and better grasp the research hotspots. As depicted in Figure 7A, the frequency of the leading 10 keywords includes burnout, COVID-19, nurses, stress, impact, depression, anxiety, mental health, prevalence, and resilience. Based on these findings, it is evident that the top 10 keywords in terms of frequency are closely aligned with our research focus. According to the temporal analysis of the keywords, among the 7 clusters, #0 compassion fatigue emerged as an early research focal point in this field and has been extensively investigated until recent years. Additionally, it is evident that research on caregiver burnout in the post-pandemic era has been diminishing. In fact, numerous articles have examined the comparison of job burnout among nursing staff during and after the epidemic, however, further investigation is warranted regarding the mental health of nursing staff in the post-epidemic era.^[45]^

There are several noteworthy limitations in this study. Firstly, our selection of WoSCC as the sole database may result in the omission of relevant papers from other databases (Scopus, Pubmed, etc). However, due to constraints imposed by bibliometric software and for reasons explained in Section 2, it was challenging to merge multiple databases for analysis. Secondly, there is a possibility of overlooking significant non-English papers, leading to research bias and diminished credibility. Lastly, given the continuous updates to databases, recently published high-quality articles might be underestimated due to inadequate citation records.

## 5. Conclusions

In summary, this is the first comprehensive analysis of publications related to nursing burnout in the context of COVID-19 from 2020 to 2023 through bibliometrics. Our results show the COVID-19 pandemic has certainly had a significant impact on the occupational burnout of nurses. Currently, the United States holds a dominant position in this field. It is crucial to enhance international transboundary cooperation among institutions and countries, particularly for developing nations. Nursing-related articles warrant heightened attention, and nursing journals should strive for more robust and substantive contributions. Naturally, the issue of nursing burnout merits ongoing concern.

## Acknowledgments

We would like to thank Jing Lu for the linguistic assistance during the preparation of this manuscript.

## Author contributions

**Conceptualization:** Da-long Wan.

**Data curation:** Ya-Xi Lu.

**Methodology:** Da-long Wan.

**Visualization:** Ya-Xi Lu.

**Writing – original draft:** Ya-Xi Lu.

**Writing – review & editing:** Da-long Wan.

## References

[1] Maslach C Schaufeli WB Leiter MP. Job burnout. Annu Rev Psychol. 2001;52:397–422. 11148311 10.1146/annurev.psych.52.1.397

[2] WHO. Burn-out an “occupational phenomenon”: international classification of diseases. 2019. https://www.who.int/mental_health/evidence/burn-out/en/. Accessed September 5, 2024.

[3] Woo T Ho R Tang A Tam W. Global prevalence of burnout symptoms among nurses: a systematic review and meta-analysis. J Psychiatr Res. 2020;123:9–20. 32007680 10.1016/j.jpsychires.2019.12.015

[4] Marair SA Slater N. Middle Eastern nurses’ views/experiences of work and well-being with the support measures during past disease outbreaks and COVID-19: a qualitative systematic review. BMC Nurs. 2023;22:230. 37400825 10.1186/s12912-023-01343-4 PMC10316637

[5] Hoff T Carabetta S Collinson GE. Satisfaction, burnout, and turnover among nurse practitioners and physician assistants: a review of the empirical literature. Med Care Res Rev. 2019;76:3–31. 28901205 10.1177/1077558717730157

[6] World Health Organisation. Health workforce requirements for universal health coverage and the sustainable development goals. Human Resources for Health Observer, 17. 2016.

[7] Davidson JE Proudfoot J Lee K Zisook S. Nurse suicide in the United States: analysis of the center for disease control 2014 national violent death reporting system dataset. Arch Psychiatr Nurs. 2019;33:16–21. 31711588 10.1016/j.apnu.2019.04.006 PMC7927355

[8] Valdes-Elizondo GD Alvarez-Maldonado P Ocampo-Ocampo MA Hernandez-Rios G Reding-Bernal A Hernandez-Solis A. Burnout symptoms among physicians and nurses before, during and after COVID-19 care. Rev Lat Am Enfermagem. 2023;31:10. 10.1590/1518-8345.6820.4047 PMC10631294 37937599

[9] Zeng L Liu D Liang X. The levels and influencing factors of compassion fatigue among new nurses during the COVID-19 pandemic. J Nurs Manag. 2023;2023:436284. 10.1155/2023/4362841 PMC11919100 40225694

[10] Vogt KS Simms-Ellis R Grange A. Critical care nursing workforce in crisis: a discussion paper examining contributing factors, the impact of the COVID-19 pandemic and potential solutions. J Clin Nurs. 2023;32:7125–34. 36823696 10.1111/jocn.16642

[11] Rizzo A Yildirim M Oztekin GG. Nurse burnout before and during the COVID-19 pandemic: a systematic comparative review. Front Public Health. 2023;11:1225431. 37732086 10.3389/fpubh.2023.1225431 PMC10507882

[12] van Mol M de Veer M de Pagter A. Vitality, resilience and the need for support among hospital employees during the COVID-19 pandemic: study protocol of a mixed-methods study. BMJ Open. 2021;11:e049090. 10.1136/bmjopen-2021-049090 PMC8503920 34625413

[13] Zheng Y Tang PK Lin GH. Burnout among healthcare providers: its prevalence and association with anxiety and depression during the COVID-19 pandemic in Macao, China. PLoS One. 2023;18:e0283239. 36928867 10.1371/journal.pone.0283239 PMC10019613

[14] Wang J Shahzad F. A visualized and scientometric analysis of health literacy research. Front Public Health. 2021;9:811707. 35155357 10.3389/fpubh.2021.811707 PMC8830295

[15] Chen C Song M. Visualizing a field of research: a methodology of systematic scientometric reviews. PLoS One. 2019;14:e0223994. 31671124 10.1371/journal.pone.0223994 PMC6822756

[16] Chen Z Ding C Gu Y. Association between gut microbiota and hepatocellular carcinoma from 2011 to 2022: bibliometric analysis and global trends. Front Oncol. 2023;13:1120515. 37064156 10.3389/fonc.2023.1120515 PMC10098157

[17] Wu Y Wang J Luo C. A comparison of burnout frequency among oncology physicians and nurses working on the frontline and usual wards during the COVID-19 epidemic in Wuhan, China. J Pain Symptom Manage. 2020;60:e60–5. 32283221 10.1016/j.jpainsymman.2020.04.008 PMC7151285

[18] Spitzer RL Kroenke K Williams JB Lowe B. A brief measure for assessing generalized anxiety disorder: the GAD-7. Arch Intern Med. 2006;166:1092–7. 16717171 10.1001/archinte.166.10.1092

[19] Pappa S Ntella V Giannakas T Giannakoulis VG Papoutsi E Katsaounou P. Prevalence of depression, anxiety, and insomnia among healthcare workers during the COVID-19 pandemic: a systematic review and meta-analysis. Brain Behav Immun. 2020;88:901–7. 32437915 10.1016/j.bbi.2020.05.026 PMC7206431

[20] Maunder RG Lancee WJ Balderson KE. Long-term psychological and occupational effects of providing hospital healthcare during SARS outbreak. Emerg Infect Dis. 2006;12:1924–32. 17326946 10.3201/eid1212.060584 PMC3291360

[21] Maslach C Jackson SE. The measurement of experienced burnout. J Organ Behav. 1981;2:99–113.

[22] Lega I Nistico L Palmieri L. Psychiatric disorders among hospitalized patients deceased with COVID-19 in Italy. EClinicalMedicine. 2021;35:100854. 33907730 10.1016/j.eclinm.2021.100854 PMC8062162

[23] Lai J Ma S Wang Y. Factors associated with mental health outcomes among health care workers exposed to coronavirus disease 2019. JAMA Netw Open. 2020;3:e203976. 32202646 10.1001/jamanetworkopen.2020.3976 PMC7090843

[24] Galanis P Vraka I Fragkou D Bilali A Kaitelidou D. Nurses’ burnout and associated risk factors during the COVID-19 pandemic: a systematic review and meta-analysis. J Adv Nurs. 2021;77:3286–302. 33764561 10.1111/jan.14839 PMC8250618

[25] Barello S Palamenghi L Graffigna G. Burnout and somatic symptoms among frontline healthcare professionals at the peak of the Italian COVID-19 pandemic. Psychiatry Res. 2020;290:113129. 32485487 10.1016/j.psychres.2020.113129 PMC7255285

[26] Kim JS Choi JS. Factors influencing emergency nurses’ burnout during an outbreak of middle east respiratory syndrome coronavirus in Korea. Asian Nurs Res (Korean Soc Nurs Sci). 2016;10:295–9. 28057317 10.1016/j.anr.2016.10.002 PMC7104920

[27] Adams JG Walls RM. Supporting the health care workforce during the COVID-19 global epidemic. JAMA. 2020;323:1439–40. 32163102 10.1001/jama.2020.3972

[28] Khalid I Khalid TJ Qabajah MR Barnard AG Qushmaq IA. Healthcare workers emotions, perceived stressors and coping strategies during a MERS-CoV outbreak. Clin Med Res. 2016;14:7–14. 26847480 10.3121/cmr.2016.1303 PMC4851451

[29] Zhu N Zhang D Wang W. A novel coronavirus from patients with pneumonia in China, 2019. N Engl J Med. 2020;382:727–33. 31978945 10.1056/NEJMoa2001017 PMC7092803

[30] Li Q Guan X Wu P. Early transmission dynamics in Wuhan, China, of novel coronavirus-infected pneumonia. N Engl J Med. 2020;382:1199–207. 31995857 10.1056/NEJMoa2001316 PMC7121484

[31] Huang C Wang Y Li X. Clinical features of patients infected with 2019 novel coronavirus in Wuhan, China. Lancet. 2020;395:497–506. 31986264 10.1016/S0140-6736(20)30183-5 PMC7159299

[32] Holshue ML DeBolt C Lindquist S. First case of 2019 novel coronavirus in the United States. N Engl J Med. 2020;382:929–36. 32004427 10.1056/NEJMoa2001191 PMC7092802

[33] Lai JB Hu SH. China sets up the specialised committee of mental health rehabilitation. Lancet Psychiatry. 2020;7:20. 31860454 10.1016/S2215-0366(19)30481-X

[34] de Oliveira DG da Cunha Reis A de Melo Franco I Braga AL. Exploring global research trends in burnout among nursing professionals: a bibliometric analysis. Healthcare (Basel). 2021;9:1680. 34946406 10.3390/healthcare9121680 PMC8700827

[35] Barragan Martin AB Molero Jurado MDM Perez-Fuentes MDC Simon Marquez MDM Sisto M Gazquez Linares JJ. Published research on burnout in nursing in Spain in the last decade: bibliometric analysis. Healthcare (Basel). 2020;8:478. 33198176 10.3390/healthcare8040478 PMC7711533

[36] Shen Z Hu J Wu H. Global research trends and foci of artificial intelligence-based tumor pathology: a scientometric study. J Transl Med. 2022;20:409. 36068536 10.1186/s12967-022-03615-0 PMC9450455

[37] Yao YG Zhang Y Zheng YT. An“impact”in publishing. Zool Res. 2019;40:239–40. 31310062 10.24272/j.issn.2095-8137.2019.040 PMC6680127

[38] Garfield E. The history and meaning of the journal impact factor. JAMA. 2006;295:90–3. 16391221 10.1001/jama.295.1.90

[39] Beall J. Predatory publishers are corrupting open access. Nature. 2012;489:179. 22972258 10.1038/489179a

[40] Tomlinson OW. Predatory publishing in medical education: a rapid scoping review. BMC Med Educ. 2024;24:33. 38183007 10.1186/s12909-024-05024-x PMC10770935

[41] Valizadeh L Zamanzadeh V Alizadeh S Namadi Vosoughi M. Promoting evidence-based nursing through journal clubs: an integrative review. J Res Nurs. 2022;27:606–20. 36405802 10.1177/17449871211022799 PMC9669933

[42] Bruyneel A Bouckaert N Maertens de Noordhout C. Association of burnout and intention-to-leave the profession with work environment: a nationwide cross-sectional study among Belgian intensive care nurses after two years of pandemic. Int J Nurs Stud. 2023;137:104385. 36423423 10.1016/j.ijnurstu.2022.104385 PMC9640385

[43] Bruyneel A Smith P Tack J Pirson M. Prevalence of burnout risk and factors associated with burnout risk among ICU nurses during the COVID-19 outbreak in French speaking Belgium. Intensive Crit Care Nurs. 2021;65:103059. 33875341 10.1016/j.iccn.2021.103059 PMC9759739

[44] Soares JP Lopes RH Mendonca PBS Silva CRDV Rodrigues CCFM Castro JL. Use of the maslach burnout inventory among public health care professionals: scoping review. JMIR Ment Health. 2023;10:e44195. 37477960 10.2196/44195 PMC10403803

[45] Mino MV Vacca A Longo R Lucisani G Solomita B Franza F. Stress work and hopelessness in mental health workers/caregivers: an observational study in pandemic and post COVID-19 pandemic. Psychiatr Danub. 2023;35(Suppl 2):266–70. 37800239

